# Role of APOA1 in the resistance to platinum-based chemotherapy in squamous cervical cancer

**DOI:** 10.1186/s12885-022-09528-x

**Published:** 2022-04-14

**Authors:** Yue He, Su-Bin Han, Yang Liu, Jing-Jing Zhang, Yu-Mei Wu

**Affiliations:** Department of Gynecological Oncology, Beijing Obstetrics and Gynecology Hospital, Capital Medical University, Beijing Maternal and Child Health Care Hospital, Dongcheng District, Qi-he-lou street No.17, Beijing, 100006 China

**Keywords:** Cervical cancer, Chemotherapy resistance, Apolipoprotein A1, Platinum,Proteomics

## Abstract

**Background:**

To investigate the mechanism by which apolipoprotein A1 (APOA1) enhances the resistance of cervical squamous carcinoma to platinum-based chemotherapy.

**Methods:**

Two cervical squamous carcinoma cell lines (SiHa and Caski) overexpressing APOA1 were constructed, treated with carboplatin, and compared to normal control cells.

**Results:**

In both SiHa and Caski cell lines, the clone-forming ability of CBP-treated cells was lower than that of untreated cells, and the change in the number of clones of overexpressing cells was lower than that of normal control cells (*p* < 0.05), indicating that APOA1 overexpression enhanced chemoresistance. A screen for APOA1 downstream proteins affecting platinum-based chemoresistance using Tandem Mass Tag revealed 64 differentially expressed proteins in SiHa cells, which were subjected to Gene Ontology (annotation, Kyoto Encyclopedia of Genes and Genomes enrichment, subcellular localization, structural domain annotation and enrichment, clustering, and interaction network analyses. Sixty-four differentially expressed proteins matching cancer-relavent association terms were screened and parallel response monitoring identified 29 proteins as possibly involved in the mechanism of platinum-based chemoresistance.

**Conclusions:**

Our analysis suggested that the mechanism may involve numerous regulatory pathways, including promoting tumor growth via the p38 MAPK signaling pathway through STAT1, promoting tumor progression via the PI3K signaling pathway through CD81 and C3, and promoting resistance to platinum-based chemotherapy resistance through TOP2A. The present study aimed to preliminarily explore the function and mechanism of APOA1 in platinum-based chemoresistance in cervical cancer, and the detailed mechanism needs to be further studied.

## Introduction

Cervical cancer is a common malignant tumor of the female reproductive system. One of the main treatments for advanced and recurrent cervical cancer is chemotherapy, and platinum-based regimens are first-line chemotherapeutics [1]. Resistance to chemotherapy is a key factor affecting treatment outcomes for advanced and recurrent cervical cancer.

Apolipoprotein A1 (APOA1) is the main structural protein in high-density lipoprotein (HDL), mainly involved in reverse cholesterol transport. Several studies have found that APOA1 plays important roles in tumor growth, angiogenesis, and invasion, and metastasis [2]. Although a few studies on APOA1 and tumor chemotherapy have been reported, the mechanism underlying the role of APOA1 in platinum-based chemotherapy resistance in cervical cancer has not been reported. We previously constructed cervical cancer SiHa cell lines stably overexpressing the *APOA1* gene using a lentiviral vector [3] and demonstrated that APOA1 is a cervical cancer drug resistance-associated protein involved in resistance to chemotherapy, especially platinum-based chemotherapy, in cervical cancer [4].

We have performed cloning, MTT and TUNEL test to find the function of APOP1 in the resistance to platinum-based chemotherapy in squamous cervical cancer (SiHa and Caski). We find that APOA1 may play the role in chemotherapy resistance by inhibit cell cloning. A screen for APOA1 downstream proteins affecting platinum-based chemoresistance using TMT revealed 64 differentially expressed proteins in SiHa cells, Sixty-four differentially expressed proteins matching cancer-relavent association terms were screened and parallel response monitoring (PRM) identified 29 proteins proteins as possibly involved in the mechanism of platinum-based chemoresistance. The present study aimed to preliminarily explore the function and mechanism of APOA1 in platinum-based chemoresistance in cervical cancer, and the detailed mechanism needs to be further studied.

## Materials and methods

### Materials

Human cervical squamous carcinoma cell lines SiHa was purchased from the Type Culture Collection of the Chinese Academy of Sciences(Shanghai, China)and Caski was purchased from the American Type Culture Collection (ATCC, Manassas, VA, USA). The following materials were used in this study: APOA1-overexpression (OE) and control lentivirus-transfected (negative control, NC) (GeneChem,Shanghai, China), DMEM (HyClone, Utah, USA), puromycin(Thermo Fisher Scientific, Waltham, USA), fetal bovine serum (Four Seasons Green, Hangzhou, China), carboplatin (CBP) (CST, Boston, USA), Triton X-100 (Sigma, St. Louis, USA), TMR red (Roche, Basel, Switzerland), Transwell kit (Corning, New York, USA), GIEMSA staining solution (Sigma, St. Louis, USA) and MTT(Genview, Genview, USA).

## Methods

### Screening stable lentivirus-transfected cell lines

APOA1-overexpression (OE) and control lentivirus-transfected (negative control, NC) with puromycin were purchased from GeneChem (Shanghai, China). SiHa and Caski cells with lentivirus with puromycin were co-cultured in DMEM containing 10% fetal bovine serum at 37℃ in a 5% CO2 incubator. The medium was changed every 2–3 days with 3.00 μg/ml puromycin, until all SiHa and Caski cells in the Blank group died, finally SiHa and Caski cell lines stably overexpressing APOAl were screened. Cells in logarithmic growth phase were used in the experiments.

### Clone formation assay

SiHa and Caski OE and NC cells were collected, trypsinized, and counted; then, the cell suspensions were diluted and inoculated into 6-well plates at a concentration of 800 cells per well. The cells were cultured in a cell culture incubator for 14 days, until visible cell clones appeared. The NC/OE + CBP groups were placed in complete medium containing carboplatin (CBP) (30 μg/ml), and incubated for 48 h. Clones were counted with the naked eye, and the number of clones with > 50 cells were counted under a microscope.

### MTT assay

SiHa and Caski OE and NC cells were collected, trypsinized, and counted. The density of the cell suspensions was adjusted to 2 × 10^3^ cells/ml, and the cells were inoculated into 96-well plates and incubated for 1–5 days, the carboplatin groups were treated with carboplatin (30 μg/ml) for 48 h (day 4 and 5). After incubation, the medium was discarded, and 20 μL of MTT (5 mg/mL) was added to each well. Then, 100 μL of DMSO was added to dissolve the formazan crystals, and the OD at 490 nm was measured with a microplate reader. Finally, the relative cell viability was calculated based on the OD, and a growth curve was generated.

### TUNEL assay

SiHa and Caski OE and NC cells were collected, trypsinized, and counted. The cells were then washed, placed in complete medium containing carboplatin (CBP) 30 ug/ml, and incubated for 48 h. Cells were fixed with 4% paraformaldehyde in PBS solution (pH 7.4) at 15–25 °C for 1 h and then washed with PBS. Cells were permeabilized using a sodium citrate solution containing 0.1% Triton X-100 and incubated in an ice bath (2–8 °C) for 2 min. Then, 50 μl of prepared DNase I was added, and the cells were incubated at 20 °C for 10 min. TUNEL reaction mix was prepared by mixing 50 μl of TdT with 450 μl of fluorescein-labeled dUTP solution. The prepared TUNEL reaction mix (50 μl) was added to each specimen, which was incubated at 37 °C for 1 h. The negative control contained fluorescein-labeled dUTP solution only. Nuclei were stained with 50 μl of 5 μg/ml DAPI for 5 min at 20℃ and rinsed thrice in PBS. Finally, TUNEL and DAPI-labeled cells were counted separately under a fluorescence microscope.

### Tandem Mass Tag (TMT) screening of ApoA1 downstream related proteins

We chose the common squamous cervical cell type SiHa cells for further tests. The samples were divided into four group (OE, NC, NC + CBP and OE + CBP), four group labeled with different isotopes and three independent trials were conducted. The types of isotope used in the analysis were following: NC1-126, NC2-127 N, NC3-127C, NC + CBP1-128 N, NC + CBP2-128C, NC + CBP3-131 N, OE1-131C, OE2-132 N, OE3-132C, OE + CBP1-133 N, OE + CBP2-133C, OE + CBP3-134 N. Biological analyses included *Gene Ontology (*GO) term analysis (http://geneontology.org/), Kyoto Encyclopedia of Genes and Genomes (KEGG) pathway annotation and enrichment analysis, subcellular localization analysis, domain annotation and enrichment analysis, and clustering and interaction network analysis. Different expressed proteins between groups that met the screening criteria, i.e., expression > 1.2 times higher or lower and a *p* value less than 0.05 by t-test, were regarded as differentially expressed proteins.

#### *Protein sampling and electrophoresis *[5]

Cell lysate samples were mixed with SDT buffer (4% SDS, 100 mM DTT, 150 mM Tris–HCl, pH 8.0), sonicated, and then boiled for 15 min. After centrifugation at 14,000 × *g* for 40 min, the protein concentration in the supernatant was quantified using the BCA Protein Assay Kit (P0012; Beyotime, Shanghai, China). The sample was stored at -20 °C until use.

#### Filter-aided sample preparation (FASP) [5]

Samples containing 200 μg of protein were mixed with 30 μl of SDT buffer. Then, the detergent, DTT, and other low-molecular-weight components were removed by repeated ultrafiltration (30 kDa; Sartorius) into UA buffer (8 M Urea, 150 mM Tris–HCl, pH 8.5). Then, 100 μl of iodoacetamide (100 mM in UA buffer) was added to block reduced cysteine residues, and the samples were incubated for 30 min in the dark. The filters were washed thrice with 100 μl of UA buffer and then twice with 100 μl of 0.1 M TEAB buffer. Finally, the proteins were digested with 4 μg of trypsin (Promega, Madison, USA) in 40 μl of 0.1 M TEAB buffer overnight at 37 °C, and the resulting peptides were collected as a filtrate. The peptide content was estimated by measuring UV light spectral density at 280 nm using an extinction coefficient of 1.1 in a 0.1% (g/l) solution, which was calculated based on the frequency of tryptophan and tyrosine residues in vertebrate proteins.

#### TMT Labeling

An aliquot of each sample (containing 100 μg of peptides) was labeled with TMT reagent (TMT 10plex™ Isobaric Label Reagent Set) according to the manufacturer’s instructions (Thermo Fisher Scientific, Waltham, USA).

#### Peptide fractionation using reversed phase (RP) chromatography

TMT-labeled peptides were fractionated by RP chromatography using an Agilent 1260 Infinity II HPLC system. The peptide mixture was diluted with buffer A (10 mM HCOONH_4_, 5% ACN, pH 10.0) and loaded onto a XBridge Peptide BEH C18 Column (130 Å, 5 µm, 4.6 mm × 100 mm). The peptides were eluted at a flow rate of 1 ml/min with a gradient of 0–7% buffer B (10 mM HCOONH_4_, 85% ACN, pH 10.0) for 5 min, 7–40% buffer B from 5 to 40 min, 40–100% buffer B from 45 to 50 min, and 100% buffer B for 50 to 65 min. Elution was monitored at 214 nm based on the UV light trace, and fractions were collected every 1 min from 5 to 50 min. The collected fractions were combined into 10 fractions and dried via vacuum centrifugation at 45 °C.

#### Easy nLC mass spectrometry analysis

Each fraction was subjected to nanoLC-MS/MS analysis. Each peptide mixture was loaded onto a C18-reversed phase analytical column (Acclaim PepMap RSLC 50 μm × 15 cm, nano viper, P/N164943; Thermo Fisher Scientific) in buffer A (0.1% formic acid) and separated with a linear gradient of buffer B (80% acetonitrile and 0.1% formic acid) at a flow rate of 300 nl/min: 6% buffer B for 3 min, 6–28% buffer B for 42 min, 28–38% buffer B for 5 min, 38–100% buffer B for 5 min, and 100% buffer B for 5 min.

#### Data analysis

MS/MS raw files were processed using the MASCOT engine (version 2.6; Matrix Science, London, UK) in Proteome Discoverer 2.2 and searched against the database *Uniprot_HomoSapiens_20367_20200226*. The search parameters included trypsin as the enzyme used to generate peptides and a maximum of two missed cleavages. A precursor mass tolerance of 10 ppm was specified as well as a 0.05 Da tolerance for MS2 fragments. Except for TMT labels, carbamidomethyl (C) was set as a fixed modification. Variable modifications were Oxidation (M) and Acetyl (Protein N-term). A peptide and protein false discovery rate of 1% was enforced using a reverse database search strategy. Proteins with a fold change greater than 1.2 and a *p* value less than 0.05 (Student’s *t*-test) were considered differentially expressed.

### Bioinformatics analyses

#### GO annotation

First, all protein sequences were aligned to a database downloaded from NCBI (ncbi-blast-2.2.28 + -win32.exe), and only the top 10 sequences with an E-value ≤ 1 × 10^–3^ were included in the analysis. Second, the GO term (database version: go_201504.obo) for the sequence with the top Bit-Score as determined by Blast2GO [6] was selected. Then, the annotation from the GO terms for the proteins was completed using Blast2GO Command Line. After simple annotation, InterProScan [7] was used to search the EBI database by motif, and the functional information for the motifs was added to the proteins to improve the annotation. Next, the annotation and connections between GO terms were further improved using ANNEX. Fisher’s exact test was used to enrich GO terms by comparing the number of differentially expressed proteins and total proteins correlated to the GO terms.

#### KEGG annotation [8]

Pathway analysis was performed using the KEGG database. Fisher’s exact test was used to identify the significantly enriched pathways by comparing the number of differentially expressed proteins and total proteins in each pathway.

#### Subcellular localization analysis

We used Wolf PSORT [9] to predict the location of different proteins. Wolf PSORT is a tool that is commonly used to predict the subcellular localization of proteins (https://wolfpsort.hgc.jp/). The program transforms protein sequences into digital location features based on sorting signals, the amino acid composition, and functional motifs. Then, the k-nearest neighbor classifier is used to predict their subcellular localization.

#### Domain annotation and enrichment analysis

The InterPro database integrates the functions of protein sequence family classification with domain and special site prediction. We used this database to annotate the functional domains of proteins of interest. Fisher’s exact test was used to compare the distribution of different proteins in the total protein set to evaluate the significance of the enrichment of a specific functional domain.

#### Clustering

For the first step of cluster analysis, the quantitative information for the target protein set was normalized. Then, Matplotlib was used to classify the two dimensions of sample and protein expression (distance algorithm: Euclidean, connection method: average link). Finally, a hierarchical clustering heat map was generated.

#### Protein–protein interaction network

To analyze the PPI networks, the gene symbols of the target protein were obtained from the database containing the target protein sequences, and these gene symbols were used in intAct (http://www.ebi.ac.uk/intact/main.xhtml). The direct and indirect interactions between the target proteins were found in the database. The interaction network was generated and analyzed using Cytoscape software (version: 3.2.1).

### Analysis of IPA-related words

We choose the keyword according to literatures about chemotherapy resistance pathway and progression of cancer. The IPA analysis was performed with the following keywords as input: JAK stat, Notch signaling, p38 MAPK signaling, PI3K signaling in B lymphocytes, tumor progression, chemotherapy, relapse, drug resistance, and metastasis.

### Identification of AOPA1 downstream proteins by Parallel Response Monitoring (PRM)

#### Ion screening of PRM peptides

Using Proteome Discoverer 2.1 (Thermo Fisher Scientific) software [10], the original spectrum file generated by Q Exactive was transformed into an MGF file, which was submitted to the Mascot 2.6 server for database retrieval using the built-in tool of the software. The database used was *UniProt_HomoSapiens_20367_20200226* (http://www.uniprot.org). Based on the results of the analysis, the unique peptides of the target proteins were screened, and information, such as the mass charge ratio, number of charges, and retention time, were obtained and imported into the inclusion list.

#### Quantitative identification of PRM

Mass spectrometry was performed using an Easy nLC according to the manufacturer’s instructions. The mass spectrum parameters were as follows: for Full-MS. scan range (m/z) 350–1800, resolution = 70,000, AGC target = 3e6, maximum injection time = 50 ms; for PRM, resolution = 17,500, AGC target = 2e5, maximum injection time = 45 ms, Loop count = 10, Isolation window = 2 m/z, and NCE = 27%.

### Statistical methods

SPSS 20.0 was used for all statistical analyses and for generating graphics. Measurement data were expressed as mean ± standard deviation (x ± s). Statistical *t*-test was used to compare the means between two groups, ANOVA was used to compare the means among multiple groups, and the chi square test was used to compare rates between groups, A *p* value less than 0.05 was considered statistically significant. Three independent trials were conducted in each assay. The dose of carboplatin used in clone formation assay, MTT assay and TUNEL was 30μg/ml which was according to the pre-experiment. We preformed 3–5 concentration gradients in pre-experiment, and choose the most suitable one for the formal experiment.

## Results

### Effects of APOA1 overexpression on cervical squamous carcinoma cells after carboplatin treatment

#### Effect on clone formation ability

**Fig. 1:**
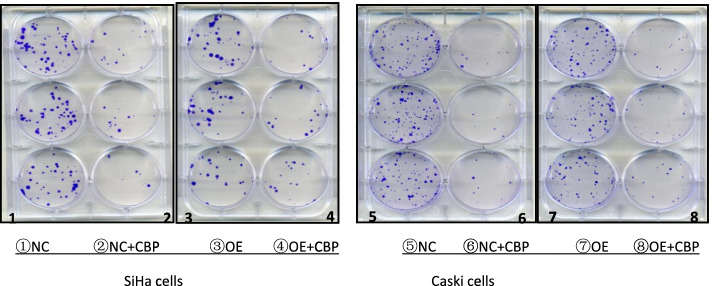
Clone formation assay of APOA1-overexpressing SiHa and Caski cells

**Fig. 2:**
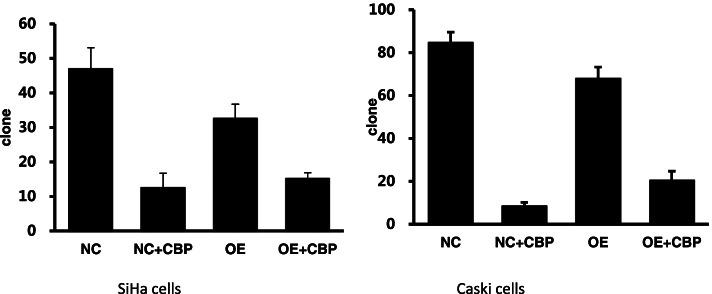
Clone formation histogram of APOA1-overexpressing SiHa and Caski cells. NC: SiHa or Caski cells with control lentivirus, OE: SiHa or Caski APOA1-overexpressing cells, NC + CBP: SiHa or Caski cells with control lentivirus treated with CBP for 48 h, OE + CBP: SiHa or Caski APOA1-overexpressing cells treated with CBP for 48 h

To investigate the effect of APOA1 overexpression on the clone formation ability of cervical squamous carcinoma SiHa and Caski cells treated with carboplatin, the colony forming ability of both normal cells (NC) and cells overexpressing APOA1 (OE) was observed in the presence and absence of carboplatin using a plate colony assay. Cells were treated with carboplatin (30 μg/ml) for 48 h, and the number of colonies was counted after 12 days of incubation. The results showed that in Caski cells, the number of clones was 85 ± 5 in the NC group, 68 ± 5 in the OE group, 9 ± 2 in the NC + carboplatin group, and 21 ± 4 in the OE + carboplatin group. In SiHa cells, the number of clones was 47 ± 6 in the NC group, 33 ± 4 in the OE group, 13 ± 4 in the NC + carboplatin group, and 15 ± 2 in the OE + carboplatin group. Statistical *t*-test was used to compare the means between two groups. Compared with NC group, the number of cell clones in OE group decreased slightly (*p* < 0.05); compared with NC + CBP group, the number of cell clones in OE + CBP group increased (*p* < 0.05). Then, in both cell lines, the change in the number of clones(the number of clones in OE -the number of clones in OE + CBP vs the number of clones in NC- the number of clones in NC + CBP) following administration of chemotherapy was smaller in the OE group than in the NC group (*p* < 0.05) (Figs. 1 and 2).

#### Effect on cell proliferation

**Fig. 3:**
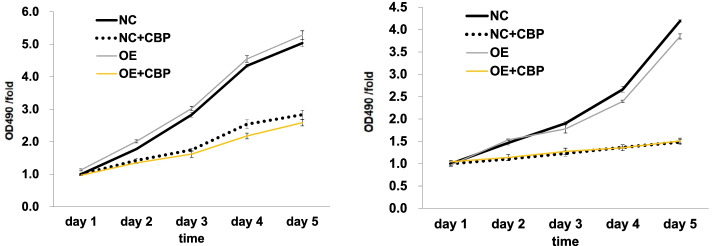
MTT assay to assess the effect of APOA1 overexpression on the proliferation of SiHa and Caski cells. NC: SiHa or Caski cells with control lentivirus, OE: SiHa or Caski APOA1-overexpressing cells, NC + CBP: SiHa or Caski cells with control lentivirus treated with CBP for 48 h, OE + CBP: SiHa or Caski APOA1-overexpressing cells treated with CBP for 48 h

To investigate the effect of APOA1 overexpression on the proliferation of cervical squamous carcinoma SiHa and Caski cells, NC and OE cells were incubated for 1–5 days and then treated with carboplatin (30 μg/ml) for 48 h. Relative cell viability was measured by the MTT assay. The results showed that the difference in cell proliferation between NC and OE cells was not statistically significant, with or without carboplatin (*p* > 0.05) (Fig. 3).

#### Effect on apoptosis

**Fig. 4:**
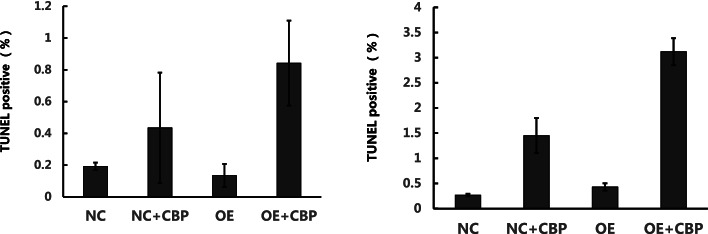
TUNEL assay to detect the effect of APOA1 overexpression on apoptosis of SiHa/Caski cells with and without CBP. NC: SiHa or Caski cells with control lentivirus, OE: SiHa or Caski APOA1-overexpressing cells, NC + CBP: SiHa or Caski cells with control lentivirus treated with CBP for 48 h, OE + CBP: SiHa or Caski APOA1-overexpressing cells treated with CBP for 48 h

To investigate the effect of APOA1 overexpression on the apoptosis of SiHa and Caski cells induced by carboplatin, NC and OE cells were treated with CBP (30 μg/ml) for 48 h. Then, apoptosis was assessed by the TUNEL method, compared with NC group, there was no significant change in OE group, and there was no significant change between OE + CBP group and NC + CBP group (Although *p* < 0.05, the TUNEL positive % value < 5%, indicating that apoptosis was basically not detected). The results are shown in Fig. 4.

### Screen for differentially expressed proteins downstream of APOA1 that involved in platinum-based chemoresistance and bioinformatics analysis

#### We chose SiHa cells for further protein screening and identified

Proteins were labeled using TMT and analyzed using tandem mass spectrometry (MS/MS). The obtained data were subjected to significant difference analysis, GO annotation and enrichment analysis, KEGG pathway annotation and enrichment analysis, subcellular localization analysis, structural domain annotation and enrichment analysis, clustering analysis, and interaction network analysis. After cleavage, a total of 5983 proteins were detected and included in the analyses for differentially expressed proteins downstream of APOA1.

#### Analysis of differentially expressed proteins

In the screening for differentially expressed proteins, the criteria were a greater than 1.2-fold difference in expression (for upregulated and downregulated proteins) and a *p* < 0.05 as determined by t-test. In the CBP-treated groups, there were 64 differentially expressed proteins between OE and NC cells; 35 were upregulated, and 29 were downregulated (Table 1).

**Table 1:** TMT screening of differentially expressed proteins downstream of APOA1 involved in platinum-based chemotherapy resistance

Comparison	Upregulated	Downregulated	Total
OE vs. NC	16	5	21
NC + CBP vs. NC	13	12	25
OE + CBP vs. OE	32	68	100
**OE + CBP vs. NC + CBP**	**35**	**29**	**64**

Comparisons: groups included in the protein expression comparisons: Upregulated, upregulated proteins; Downregulated, downregulated proteins; Total, all differentially expressed proteins

#### GO enrichment analysis

**Fig. 5:**
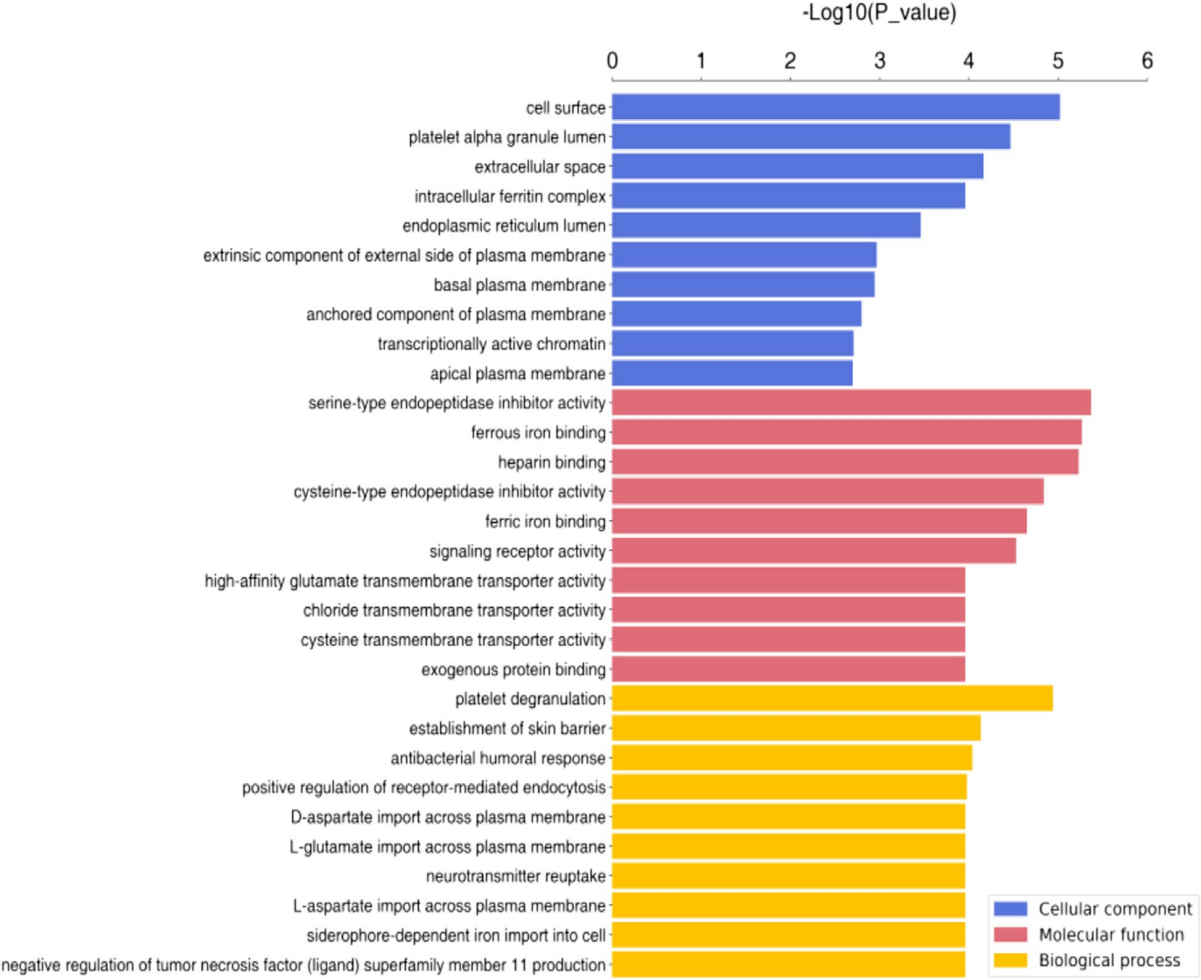
GO enrichment analysis of the differentially expressed proteins in cells overexpressing APOA1 and normal control cells treated with carboplatin. (Tag:The ordinate represents the GO function name, and the abscissa represents the enrichment significance *p*-value.)

Using GO enrichment analysis, the target proteins were classified according to biological process, molecular function, and the cellular components they reside in. The distribution of differentially expressed proteins in OE + CBP compared to NC + CBP according to each classification is shown in Fig. 5.

#### KEGG pathway enrichment analysis

**Fig. 6:**
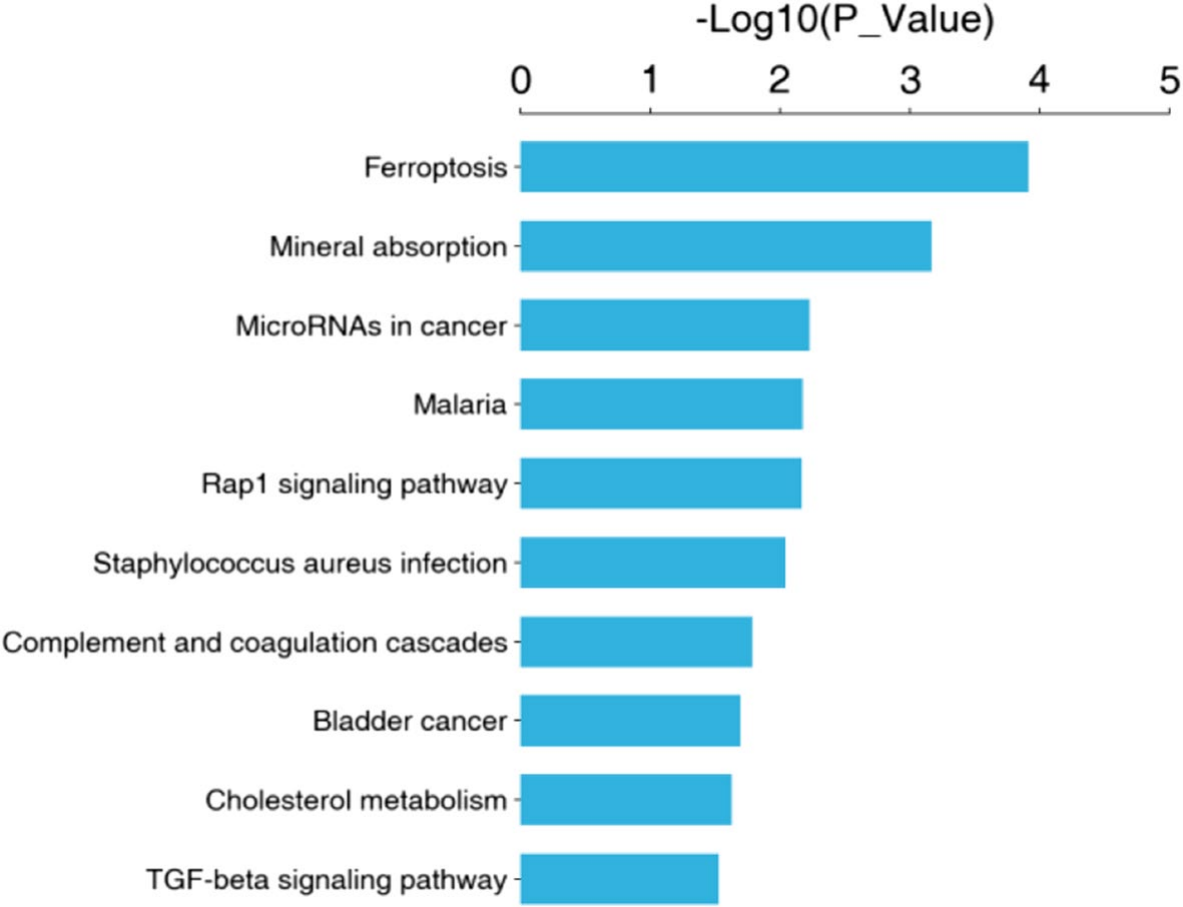
KEGG pathway enrichment analysis of the differentially expressed proteins in cells overexpressing APOA1 and normal control cells treated with carboplatin. (Tag:The ordinate represents the KEGG pathway name, and the abscissa represents the enrichment significance *p*-value)

The method used for the KEGG pathway enrichment analysis is similar to that of the GO enrichment analysis, in that the KEGG pathway was used as a unit and all qualitative proteins were used as the background. To identify significantly affected metabolic and signaling pathways, the significance of protein enrichment in each pathway was calculated using Fisher’s exact test. The OE + CBP and NC + CBP groups were compared to analyze which pathways the differentially expressed proteins downstream of APOA1 were involved in (Fig. 6).

#### Subcellular localization analysis

**Fig. 7:**
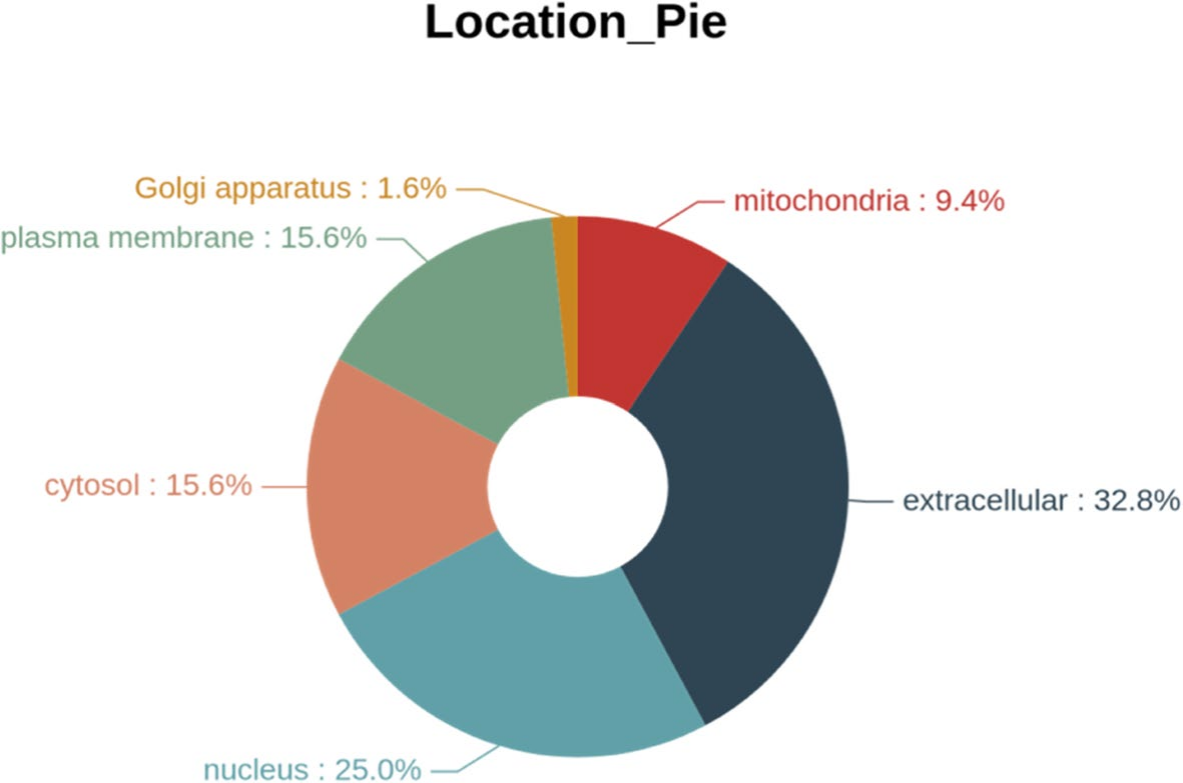
Subcellular localization of the differentially expressed proteins in cells overexpressing APOA1 and normal control cells treated with carboplatin

The subcellular localization of the differentially expressed proteins downstream of APOA1 was analyzed. The major subcellular localizations in eukaryotic cells are extracellular, cytoplasm, nucleus, cell membrane, mitochondria, Golgi apparatus, endoplasmic reticulum, peroxisomes, vesicles, cytoskeleton, vesicle membrane, chloroplasts, nucleoplasm, nuclear matrix, and ribosomes. The subcellular localization of the proteins downstream of APOA1 that were differentially expressed proteins between OE + CBP and NC + CBP were analyzed using WoLF PSORT software. The results showed that these proteins were localized to the extracellular matrix (32.8%), nucleus (25%), cytoplasm (15.6%), plasma membrane(15.6%), mitochondria (9.4%) and Golgi apparatus (1.6%) (Fig. 7).

#### Structural domain annotation and enrichment analysis

**Fig. 8:**
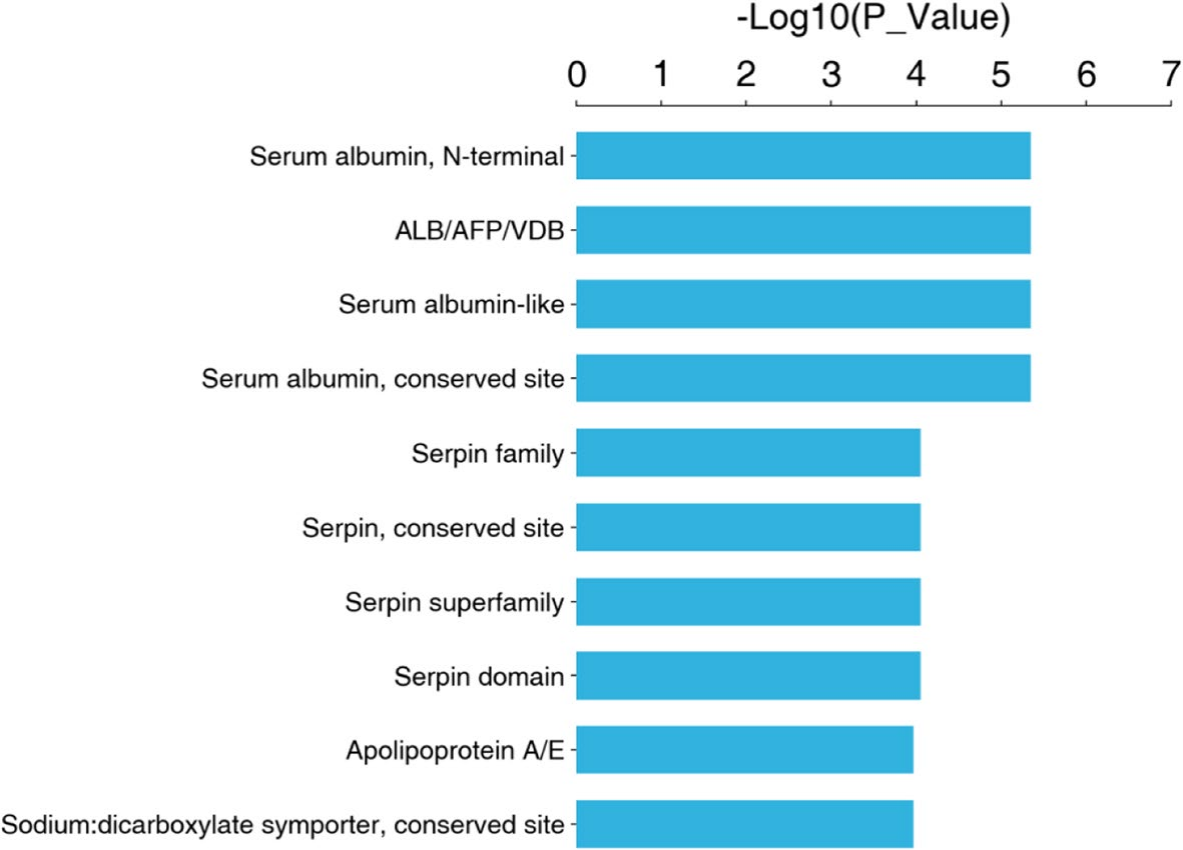
Structural domain annotation and enrichment analysis in cells overexpressing APOA1 and normal control cells treated with carboplatin

The functional structural domains of the differentially expressed proteins downstream of APOA1 between OE + CBP and NC + CBP were analyzed for enrichment using the InterPro database (see Fig. 8).

#### IPA bioinformatics analysis

We used the keywords JAK STAT, Notch signaling, p38 MAPK signaling, PI3K signaling in B lymphocytes, tumor progression, chemoresistance, relapse, drug resistance and metastasis as the input for the IPA bioinformatics analysis. Of the 64 differentially expressed proteins downstream of APOA1, 29 proteins were screened by PRM, including 10 upregulated proteins and 19 downregulated proteins, as target proteins for PRM protein identification and quantification.

### Quantitative analysis of PRM proteins

Of the 29 screened downstream proteins, which were subjected to quantitative PRM protein analysis, 13 had no statistically significant expression differences between the OE and NC groups but did have statistically significant expression differences between the OE + CBP and NC + CBP groups (Table 2 marked in bold), suggesting that these 13 proteins, which are downstream factors of APOA1, may be involved in platinum-based chemoresistance in cervical cancer. Literature searches were conducted to find APOA1 downstream proteins and mechanisms affecting resistance to platinum-based chemotherapy in cervical cancer. Our search identified the following: 1) TOP2A, CDKN2A, and APOE, which are involved in tumor growth, recurrence, and metastasis; 2) STAT1, which may be involved in metastasis of tumor through the p38 MAPK signaling pathway; 3) CD81, C3, and RAC1, which may promote tumor progression through the PI3K signaling pathway; and 4) other proteins, such as ALB, AFP, GC, LTF, SOD2, and NDRG1, which are involved in tumor metastasis.

**Table 2:** PRM identification of APOA1 downstream proteins involved in platinum-based chemotherapy resistance in cervical cancer

No	Gene name	OE/NC	*P* value	OE + CBP/NC + CBP	*P* value
**1**	CCN1	1.368	0.0011	0.906	0.0252
**2**	DCTN3	1.084	0.0391	0.809	0.0280
**3**	**C3**	**1.066**	**0.1338**	**0.646**	**0.0011**
4	APOA1	3.577	0.0005	2.645	0.0001
**5**	**APOE**	**0.863**	**0.2822**	**1.906**	**0.0002**
**6**	AHSG	1.812	0.0133	0.412	0.0121
**7**	**ALB**	**1.219**	**0.0926**	**0.468**	**0.0009**
**8**	**AFP**	**1.097**	**0.1194**	**0.418**	**0.0013**
**9**	**GC**	**1.040**	**0.1678**	**0.397**	**0.0001**
**10**	**LTF**	**1.011**	**0.9124**	**0.402**	**0.0001**
11	FTH1	1.048	0.3035	1.190	0.1577
**12**	**SOD2**	**1.020**	**0.8558**	**1.359**	**0.0146**
13	THBS1	0.748	0.0039	0.451	0.0001
14	KRT19	1.835	0.0360	1.112	0.0905
**15**	**TOP2A**	**0.942**	**0.4104**	**0.730**	**0.0079**
16	F3	1.154	0.2077	0.805	0.2458
17	JUP	1.341	0.0001	1.057	0.3250
18	MUC1	1.418	0.0153	1.372	0.0089
19	H1-5	2.016	0.0001	0.497	0.0002
20	PRM2	1.068	0.3243	0.824	0.0483
21	SERPINB5	1.120	0.1081	0.956	0.5184
**22**	**STAT1**	**1.009**	**0.8636**	**0.774**	**0.0015**
**23**	**CDKN2A**	**0.954**	**0.0892**	**0.767**	**0.0061**
**24**	**CD81**	**0.931**	**0.0978**	**0.692**	**0.0016**
25	B2M	1.375	0.0043	1.305	0.0038
**26**	**RAC1**	**0.953**	**0.4207**	**0.695**	**0.0156**
27	TNFAIP2	0.971	0.5228	1.090	0.2733
28	SLC1A5	0.610	0.0008	0.445	0.0026
**29**	**NDRG1**	**1.066**	**0.1832**	**1.653**	**0.0006**

## Discussion

### Relationship between APOA1 and tumor

As the main structural protein of HDL, APOA1 has anti-atherosclerotic, anti-inflammatory, antioxidant, and anti-endotoxic effects [11], and chronic inflammation, oxidative stress, lipids, and cholesterol are associated with tumor development. Several studies have found that serum APOA1 levels are reduced in patients with different malignancies [12, 13]. In gynecological tumors, it has been reported that APOA1 levels are significantly downregulated in patients with epithelial ovarian cancer serum, and APOA1 is a potential tumor marker for epithelial ovarian cancer [14, 15]. APOA1 was shown to inhibit tumor development, and low serum levels of APOA1 are associated with poor prognosis. In studies of advanced ovarian cancer, high APOA1 mRNA levels in body fluids were found to be an independent diagnostic factor for clinically longer overall survival (OS) [16].

In a large European prospective study, APOA1 was found to be negatively associated with the risk of colon cancer [17]. In hepatocellular carcinoma, low levels of APOA1 were associated with poorer progression free survival (PFS), thus APOA1 may be a useful predictor of recurrence in hepatocellular carcinoma [18]. In metastatic nasopharyngeal carcinoma, higher serum levels of APOA1 at pre-treatment were associated with higher OS [19]. In breast cancer, low expression of APOA1 predicted a higher risk of breast cancer development and recurrence [20]. The results of some meta-analyses suggest that low levels of APOA1 may be a poor prognostic indicator for various malignancies, including non-small cell lung cancer, gallbladder cancer, and gastric cancer [21].

### The function of APOA1 involved in tumorigenesis and development

The mechanism by which APOA1 inhibits tumors is currently unclear. Studies have shown that APOA1 exerts anti-tumor effects mainly by inhibiting tumor cell proliferation and promoting apoptosis [22, 23]. APOA1 has been reported to capture circulating tumor cells and promote tumor cell apoptosis by downregulating that MAPK pathway, thereby inhibiting tumor cell proliferation [24]. APOA1 also regulates inflammation signals through the STAT3 signaling pathway and reduces matrix metalloproteinase-9 (MMP-9) levels, which is an important factor that promotes tumor proliferation and metastasis [25]. It has also been reported that a short peptide on APOA1 regulates the phosphorylation of c-Src through the c-Src/ERK signaling pathway, thereby inhibiting tumor growth and angiogenesis [26].

APOA1 is easy to detect clinically and has great value as a marker of tumor prognosis. However, there are few studies on the correlation between APOA1 and tumor chemotherapy. Some studies have reported that APOA1 can be used as a predictive marker for platinum-based chemotherapy resistance in ovarian cancer [27]. In the field of gynecological oncology, proteomic studies have shown that APOA1 is highly expressed in platinum-based chemotherapy-resistant ovarian cancer when compared to the levels in paracancerous tissue. A screen for differentially expressed proteins in serum samples from platinum-resistant and platinum-sensitive patient groups identified APOA1 [28], indicating its association with platinum-based chemoresistance. Cruz et al. [27] searched for proteins related to the proteasomal ubiquitination resistance signaling pathway in platinum-resistant ovarian cancer cells and tissues and found that APOA1 was a significantly upregulated platinum chemoresistance-associated protein and could be used as a predictive marker of platinum chemoresistance in ovarian cancer. However, whether APOA1 is related to chemotherapy in cervical cancer has not yet been reported. In this study, we found that APOA1-overexpressing (OE) and normal control (NC) SiHa and Caski cervical squamous carcinoma cells showed smaller differences in the change of clones after administration of chemotherapy (*p* < 0.05), indicating that overexpression of APOA1 induced resistance to carbopaltin chemotherapy in cervical cancer cells and reduced the chemotherapy-induced reduction of cell cloning. In contrast, there were no significant differences in cell proliferation and apoptosis.

### The mechanism of APOA1 involved in chemoresistance

Through bioinformatic analysis and IPA bioanalysis, we found that the factors downstream of APOA1 that are involved in carboplatin chemoresistance in cervical cancer are mainly located in the extracellular matrix and nucleus and may enhance chemoresistance to carboplatin through multiple molecular mechanisms involved in cell composition, cell function, and biological processes. A literature review revealed downstream genes that may be related to chemoresistance: AFP, APOE, B2M, C3, GC, and TOPA2. Our study found seven downstream factors that may be associated with chemoresistance, LTF, SOD2, STAT1, CDKN2A, CD81, RAC1, and NDRG1 and five factors that may be associated with tumor development, CCN1, DCTN3, MUC1, H1-5, and SLC1A5. The specific mechanisms involving these genes need to be further investigated.

We identified and quantitatively validated the proteins downstream of APOA1 by PRM, which indicated that APOA1 may exert its carboplatin chemoresistance effects in cervical squamous carcinoma by 1) promoting tumor growth, recurrence, and metastasis through TOP2A, CDKN2A, and APOE; 2) regulating the p38 MAPK signaling pathway through STAT1 to promote in tumor growth and metastasis; 3) regulating the PI3K signaling pathway through CD81, C3, and RAC1 to promote tumor progression; and 4) promoting metastasis through ALB, AFP, GC, LTF, SOD2, and NDRG1. Our group investigated and validated APOA1-regulated downstream factors involved in the mechanism of platinum-based chemoresistance in cervical squamous carcinoma.

## Conclusions

This is a basic research to investigate the mechanism by which apolipoprotein A1 (APOA1) enhances the resistance of cervical squamous carcinoma to platinum-based chemotherapy. Sixty-four differentially expressed proteins matching cancer-relavent association terms were screened and parallel response monitoring (PRM) identified 29 proteins proteins as possibly involved in the mechanism of platinum-based chemoresistance. Our analysis suggested that the mechanism may involve numerous regulatory pathways, including promoting tumor growth via the p38 MAPK signaling pathway through STAT1, promoting tumor progression via the PI3K signaling pathway through CD81 and C3, and promoting resistance to platinum-based chemotherapy resistance through TOP2A.The present study aimed to preliminarily explore the function and mechanism of APOA1 in platinum-based chemoresistance in cervical cancer, and the detailed mechanism needs to be further studied.

## Data Availability

All data generated or analysed during this study are included in this published article. Px-submission accession number: PXD030885.

## Abbreviations

APOA1: Apolipoprotein A1
HDL: High-density lipoprotein
CBP: Carboplatin
TMT: Tandem Mass Tag
FASP: Filter-aided sample preparation
GO: Gene Ontology
PFS: Progression free survival
PRM: Parallel Response Monitoring
MMP-9: Matrix metalloproteinase
